# Patients’ reaction to the ethical conduct of radiographers and staff services as predictors of radiological experience satisfaction: a cross-sectional study

**DOI:** 10.1186/s12910-015-0062-4

**Published:** 2015-10-08

**Authors:** Ogbonnia Godfrey Ochonma, Charles Ugwoke Eze, Soludo Bartholomew Eze, Augustine Obi Okaro

**Affiliations:** Department of Health Administration and Management, Faculty of Health Sciences and Technology, College of Medicine, University of Nigeria, Enugu Campus, Enugu, Nigeria; Department of Medical Radiography and Radiological Sciences, Faculty of Health Sciences and Technology, College of Medicine, University of Nigeria, Enugu Campus, Enugu, Nigeria

**Keywords:** Nigeria, Patients’ satisfaction, Ethical/professional conduct, Radiographers, Patients’ perception

## Abstract

**Background:**

Patients’ satisfaction arises from their appraisal of experience in hospital services and measuring patients’ satisfaction in hospital has become a global phenomenon. To improve on patients’ satisfaction, radiographers have to imbibe the right ethical attitude in their conduct while discharging duties to patients during radiological examination. The objective of this study is to understand from the patients’ perspective the ethical conduct of radiographers and radiology nurses that constitute factors in patient satisfaction during routine radiological examination. The rationale of the study is to use the findings to improve radiological service delivery and improve on patient satisfaction.

**Methods:**

This is a cross-sectional descriptive study in which 300 respondents (outpatients) in two hospitals were surveyed to ascertain their satisfaction with the ethical conduct of radiographers and services provided by radiology nurses in the department. The data was analyzed using descriptive statistics at 95 % confidence interval for mean scores and Z-values.

**Results:**

Three hundred patients responded to the survey which comprised of 145 patients from the public hospital and 155 patients from the private hospital. Radiographers fell short in some ethical/professional conduct as in informed consent before treatment (mean = 2.95); radiographers’ not explaining his/her experience, expectation, knowledge and equipment procedure (mean = 2.98). However, they did well in some aspects including observation of professional boundaries with patients during treatment and equity in treatment for the patients during the radiological examination (mean score = 1.43). Some services provided by staff members in the department also fell short of patients’ expectation and satisfaction including explanation of what to expect during the exam (mean = 3.30), whereas they did well in their level of courtesy to patients (mean score = 4.09). There was a significant difference in the satisfaction level experienced by patients at both hospitals in favour of the private hospital.

**Conclusions:**

There is an urgent need for improved ethical/professional conduct of radiographers and general service delivery in the radiology departments of the hospitals where this investigation was carried out to enhance patient satisfaction. Government has to improve the curricular of service providers in radiology service in the university to include ethical/professional conduct and patient/provider relationship.

## Background

Patients’ satisfaction arises from their appraisal of experience in hospital services; it involves likes and dislikes which are internal and external to the patients and also relates to the extent to which general health care needs and condition-specific needs are met [1]. A Patient’s likes may show positive internal assessment while his dislikes could be appreciated by other patients in their own assessments which he/she has no control over and therefore external to him/her. What constitutes a satisfactory health service to one patient may not be for another. Measuring client or patient satisfaction has become an integral part of hospital management strategies across the globe [2, 3]. Moreover, the quality assurance and accreditation process in most countries requires that the satisfaction of clients be measured on a regular basis [2, 3]. The desired need for the measurement of patient satisfaction has been largely driven by the underlying politics of “new public management” [4, 5] and the concomitant rise in the health consumer movement, with patient satisfaction being one of the articulated goals of healthcare delivery.

Consumer perception of health care is largely ignored by health care providers in low income countries [6–8]. A search of the literature reveals that patients’ satisfaction with health services and as a matter of fact radiology in low-income countries is on the increase but less than what is obtainable in the Western countries [6–8]. Clinical radiography integrates scientific knowledge and technical skills with effective patient interaction to provide quality patient care and useful diagnostic information [9].

Effective communication between patients and providers has become imperative in radiological diagnosis [9, 10]. Radiographers, therefore, should remain sensitive to the physical and emotional needs of patients through good communication, patient-care skills and professional/ethical conduct [9, 10]. The ethical duties of radiographers include treating patients with respect and dignity and maintaining patient privacy and confidentiality at all times [9, 11]. Professional/ethical relationships and responsibilities outline trust form the basis of the relationship between a radiographer and a patient [9]. Consequently, it is expected from radiographers to behave in a manner that justifies public trust and confidence in order to uphold their professional ethics and serve both public and private interests [9, 12]. The code of conduct for radiographers makes provision for professional and ethical standards that radiographers must adhere to [12]. Radiographers have to ensure a safe working environment for the benefit of staff, patients and visitors and are also legally accountable for their professional actions and for any negligence regarding to patient’s care [9, 11]. Radiographers have at times failed or excelled in their ethical and professional conduct to patients [9]. A study [9] found that radiographers were courteous, friendly and communicated well which were sources of satisfaction but patients were not satisfied with more than necessary exposure to radiation. Staff services other than those of radiographers rendered in hospitals in the radiology departments could also constitute sources of satisfaction or dissatisfaction for the patients. A research work [13] found patients to be more satisfied with technical aspect of care, while rating down hospitalization services particularly food, resting time and information provision as sources of dissatisfaction.

Because study on patient satisfaction is a growing concept in the developing countries [6–8], the aim of this study is to investigate the ethical and professional conduct of radiographers and how it may influence patient satisfaction with radiological service in Enugu, Nigeria. The rationale behind this study is to find possible ways to improve on the ethical and professional conduct of radiographers and other staff in the department to better serve and improve the satisfaction of patients in radiology departments.

## Methods

### Study area and population

The survey took place in Enugu metropolis, Southeast Nigeria, where two hospitals—University of Nigeria Teaching Hospital, Ituku/Ozalla and Life Chart Diagnostic Centre, Abakpa-Nike, Enugu which are public and private hospitals respectively which were used for the study. The University of Nigeria Teaching Hospital (UNTH) began early in the 20th century as a standard general Hospital for Africans built by the colonial administrators. It later metamorphosed into a general hospital on the attainment of Nigeria’s independence in the 1960’s. At the end of the Nigerian civil war, the hospital had a total of 50 doctors, 10 wards, and 300 beds and a chest bay of 60 beds. Today, the situation has changed dramatically. The bed capacity of the hospital in the permanent site is over 500 beds and the number of its personnel (professional and non–professional) has increased tremendously. There are nine training schools/programmes in the hospital. Life Chart Diagnostic Centre, Abakpa-Nike, Enugu is privately owned diagnostic centre located in the middle of the town.

### Collection of data

This is a cross-sectional descriptive study in which patients who had undergone radiological examination were surveyed to understand their level of satisfaction with the ethical and professional conduct of radiographers and other staff services before, during and after the examination.

Our research conformed to the Helsinki Declaration and local legislation and ethical clearances were obtained from the University of Nigeria Teaching Hospital and Life Chart Diagnostic Centre ethical committees, all in Enugu, Nigeria.

In no particular order, patients were scheduled for examination on Mondays for the public hospital and on the average 40 patients were usually seen in a day. To allow for chance alone determine who gets included in our sample, the researchers using random sampling technique agreed that every second person seen on the clinic day would be included in our sample and interviewed. So on the average about 20 patients were interviewed at the public hospital on each clinic day. The same exercise was repeated for the private hospital that sees about 25 patients on its clinic day of Friday and as such about 13 patients were interviewed on each clinic day. The interview exercise commenced in March and ended in July of 2013.

A written consent form was provided and every patient who consented to participate in the study was asked to sign the form after reasonable explanation concerning the study was provided. Patients were provided with information concerning the risk free nature associated with partaking in the study and were assured of their confidentiality in the final analysis of the study before consenting to partake.

Respondents were provided with a 4 point Likert scale from 1(Agree), 2(Moderately Agree), 3(Disagree) to 4(Very Disagree) to enable them answer questions pertaining to the ethical and professional conduct of radiographers in the radiology department. Questions were asked on patient’s consent to treatment and right to refusal of treatment, observation of appropriate professional boundaries between patients and service providers, equity in treatment, individualized service during examination, and confidentiality of information gained from patients during examination. A pilot study was performed to validate and improve on the reliability of the questionnaire before being used for the study. First, the questions were translated into the local language-*igbo* from English language and translated back into English language from *Igbo* language to improve on its content validity. Questions that did not make sense to the respondents were either modified or discarded in entirety. This was performed until the respondents and the researchers agreed on the suitability and meaning of all the questions. The respondents were unsupervised nor watched over during their responses to the questions to minimize coercion and any influence on their responses.

### Sample size calculation

The sample size for this study was obtained using the formula: Z_1-α/2_^2^ P (1-P) which was developed by Charan et al. [14] for calculation of sample size in medical research and the findings from a previous Nigerian work [15] in which 83 % of the patients were satisfied with overall health services in the hospital.

Though the calculated sample size for the present study is 111, a total number of 300 subjects were used because of patients availability and the need to improve on the degree of reliability of generated data.

### Statistical analysis

Data analysis was achieved through the use of SPSS statistical tool. The data was entered in Epi Info and was transferred to Statistical Package for Social Sciences (SPSS 16) for analysis. The discrete data were described using frequencies and percentages, while the continuous variables were described using means and standard deviations. In addition, cross tabulations were done to establish the level of relationship or otherwise on key variables to find out the factors that influenced variables outcomes. The alpha was set at 0.05 and the researchers concluded a statistical significant relationship to exist when the P-value of the test statistics is less than or equal to 0.05.

## Results

### Table 1: Socio- demographic characteristics of the respondents

**Table 1:** Socio- demographic characteristics of the respondents

Demographic characteristics	Options	Frequency	Percent
Age	under 30	142	47.3
	31–40	61	20.3
	41–50	42	14.0
	over 50	55	18.3
Type of Centre	Public	145	48.3
	Private	155	51.7
Gender	Male	92	30.7
	Female	208	69.3
Highest level of education	no school	17	5.7
	Elementary	37	12.3
	high school	110	36.7
	college/university	115	38.3
	higher education (professional or post-graduate)	20	6.7
	literacy classes only	1	.3
Marital status	Married	63	21.0
	Separated	2	.7
	Divorced	2	.7
	married with children	122	40.7
	married without children	32	10.7
	Single	79	26.3
Length of time as radiological service patient	one month	154	51.3
	two months	11	3.7
	three to six months	27	9.0
	seven months to two years	25	8.3
	three years to 5 years	23	7.7
	five years and above	24	8.0
	can't say	36	12.0
Occupation	Student	56	18.7
	government employee	54	18.0
	private employee	41	13.7
	Unemployed	41	13.7
	self employed	39	13.0
	Retired	5	1.7
	Teaching	3	1.0
	Trader	49	16.3
	Applicant	2	.7
	Farming	8	2.7
	Rev. sister	1	.3
	Priest	1	.3
Average monthly income	no income	92	30.7
	#5,000 and below	36	12.0
	#5,000 and #20,000	51	17.0
	#21,000 and #50,000	67	22.3
	#51,000 - #100,000	39	13.0
	#101,000 - 200,000	9	3.0
	#201,000 - 400,000	3	1.0
	#401,000 - #600,000	3	1.0
Main source of payment for radiological services	Insurance	16	5.3
	self pay	261	87.0
	free medical care	12	4.0
	Children	1	.3
	Parents	6	2.0
	Pension	1	.3
	Allowance	1	.3
	NHIS	2	.7
First experience with centre	Yes	200	66.7
	No	100	33.3

As noted in Table 1; there were three (300) hundred respondents and those under thirty (30) years of age constituted the majority 142 (47.3 %). One hundred and fourty five 145(48.3 %) questionnaires were administered in the public hospital and one hundred and fifty five 155(51.7 %) questionnaires in the private hospital. There were 92 (30.7 %) male and 208(69.3 %) female respondents. Those with college/university education 115 (38.3 %) constituted the majority. Majority 92 (30.7 %) have no income presumably because they are unemployed and the means of payment for services received was self-pay as the majority 261 (87.0 %) did just that. Majority of the respondents (112 (21.0 %) were married with children and about half of them 154 (51.3 %) indicated that they have had radiological services within the last one month. Those that indicated that the radiological services they received were their first experience with their centre were in the majority 200 (66.7 %)

### Assessing the ethical/professional attitude of radiographers in the process of providing radiological service

As presented in Table 2, with the exception of principles of informed consent not being observed (mean = 2.95), radiographers’ not explaining his/her experience, expectation, knowledge and equipment procedure (mean = 2.98) (radiographers are required by their code of conduct to manage their patients’ radiological experience thoroughly through such disclosure to relax and assure them especially those of first experience with radiological examination), radiographers’ inability to disclose his/her competence and limitations where appropriate (mean = 2.83), radiographers’ inability to understand the need to build and sustain professional relationships (mean = 2.73) and the radiographers’ inability to exercise the need to engage patients in planning and evaluation of diagnostics (mean = 2.67), which were sources of dissatisfaction. The respondents indicated that the radiographers exhibited ethical conduct/professionalism in the delivery of their service. This is based on the respective mean scores < 2.5 and the overall mean score = 1.99. Furthermore, with a Z-value of 3.737 > Z_critical_ of 1.68 (2-tailed tests) and p < 0.05, this result is significant.

**Table 2:** Ethical/professional attitude of radiographers in the process of providing radiology services

Question	A (%)	MA (%)	D (%)	VD (%)	NR (%)	Mean	Std. Dev.
Was the principle of informed consent including the right of patients or their substitute to refuse service observed?	77 (25.7)	32 (10.7)	83 (27.7)	44 (14.7)	64 (21.3)	2.95	1.46
Was the dignity, privacy and autonomy of patients observed during treatment/service?	214 (71.3)	47 (15.7)	11 (3.7)	8 (2.7)	20 (6.7)	1.58	1.13
Was clear and appropriate professional boundaries maintained during service provision-patient relationship?	241 (80.3)	34 (11.3)	2 (0.7)	1 (0.3)	22 (7.3)	1.43	1.08
Were you treated equitably with other patients regardless of your tribe, ability to pay, and type of illness?	242 (80.7)	26 (8.7)	9 (3.0)	7 (2.3)	16 (5.3)	1.43	1.04
Did you receive individualised service during examination taking into account your particular physical and emotional needs, values and background	202 (67.3)	41 (13.7)	19 (6.3)	5 (1.7)	33 (11.0)	1.75	1.32
Did the radiographer observe the principle of confidentiality of information acquired through professional contact with you?	233 (77.7)	15 (5.0)	5 (1.7)	2 (0.7)	45 (15.0)	1.70	1.45
Was your examination completed on timely manner and in response to your needs?	220 (73.3)	37 (12.3)	22 (7.3)	3 (1.0)	18 (6.0)	1.54	1.09
Did the radiographer explain his/her experience, expectations, knowledge and equipment procedure to you before services	52 (17.3)	38 (12.7)	113 (37.7)	58 (19.3)	39 (13.0)	2.98	1.24
Was the radiographer competent in the performance of the radiology services	227 (75.7)	41 (13.7)	5 (1.7)	0 (0.0)	27 (9.0)	1.53	1.17
Did the radiographer take personal responsibility, use discretion and judgement in a manner that ensured your best service outcome?	225 (75.0)	39 (13.0)	9 (3.0)	1 (0.3)	26 (8.7)	1.55	1.17
Was the radiographer able to disclose his/her competence and limitations where appropriate in the process of service provision?	63 (21.0)	67 (22.3)	86 (28.7)	26 (8.7)	58 (19.3)	2.83	1.38
Did the radiographer act in your best interest during contact and service delivery?	222 (74.0)	40 (13.3)	4 (1.3)	1 (0.3)	33 (11.0)	1.61	1.27
Was the radiographer able to exercise professional duty of care by holding up to your best interest in the process of service delivery	230 (76.7)	31 (10.3)	4 (1.3)	2 (0.7)	33 (11.0)	1.59	1.28
Was the radiographer able to understand the limits of his practice and when to seek advice or refer to another professional?	167 (55.7)	50 (16.7)	9 (3.0)	5 (1.7)	69 (23.0)	2.20	1.64
Did the radiographer understand the need to build and sustain professional relationships as both an independent practitioner and collaboratively as a member of a team?	138 (46.0)	40 (13.3)	4 (1.3)	2 (0.7)	116 (38.7)	2.73	1.86
Did the radiographer exercise the need to engage you in planning and evaluating diagnostics, treatment and interventions to meet your health needs and goals	88 (29.3)	37 (12.3)	98 (32.7)	40 (13.3)	37 (12.3)	2.67	1.35
Was she/he able to make appropriate referrals as the need be in the process of care and examination	180 (60.0)	43 (14.3)	22 (7.3)	4 (1.3)	51 (17.0)	2.01	1.50
Was he/she able to demonstrate effective and appropriate skills in communicating information, advice, instruction and professional opinion to you and as the case may be, your relatives	219 (73.0)	27 (9.0)	12 (4.0)	4 (1.3)	38 (12.7)	1.72	1.37
Overall Mean Scores						1.99	
Z-Value						3.737	
*P*-value						0.000	

In this study, professional/ethical conduct of radiographers with the lowest mean values would be the better predictors of overall patient satisfaction. The mean values < 2.5 show which attitude of the radiographers the respondents agreed more with as better predictors of overall radiological service satisfaction. The greatest ethical/ professional behaviours exhibited by radiographers that were the most indicators of satisfaction for the patients are the observation of professional boundaries with patients during treatment and equity in treatment for the patients during the radiological examination which drew mean scores of 1.43 each. Other professional and ethical conduct of the radiographers that affected patients’ satisfaction were: patients receiving individualized service during examination (mean score = 1.75), radiographers’ observation of the principle of confidentiality (mean score = 1.70), timely completion of examination (mean score = 1.54), radiographers’ serving patients’ best interest (mean score = 1.59) and radiographers’ demonstration of appropriate skills in effective communication (mean score = 1.72).

### Assessing radiographers levels of professionalism

Table 3 shows that majority (84.7 %) of the respondents (patients) indicated that radiographers exhibited good professional conduct and ethics in the delivery of their service while 15.3 % of the respondents differed. This result is significant as Z-Value (8.854) > Z_critical_ (1.68) and p < 0.05.

**Table 3:** Mean level of professionalism in radiology

	Frequency	Percent	Z-value	*p*-value
Exhibited Professionalism	254	84.7	8.854	0.000
Do not Exhibit Professionalism	46	15.3		
Total	300	100.0		

### Assessing the services rendered in the radiology department and patients’ reaction

In Table 4, respondents were presented with a 5 point Likert scale ranging from 1 (very dissatisfied), 2(dissatisfied), 3(neutral), 4(satisfied) to 5 (very satisfied) and were asked to register their reaction to each item of service delivery as shown in the table. As presented in the results (Table 4), with the exception of the respondents being neutral to waiting time before procedure (mean = 3.12), explanation of what to expect during the exam (mean = 3.30) and explanation of what to expect after the exam (mean = 3.13) which are sources of dissatisfaction, the respondents are satisfied with the conducts of staff in all the other aspects of services rendered to them in the department (as the mean values > 3.5).

**Table 4:** Services rendered in the radiology department and patients’ reaction

Items	1(%)	2 (%)	3 (%)	4 (%)	5 (%)	Mean	Std. Dev.
Making an appointment	19 (6.3)	18 (6.0)	143 (47.7)	51 (17.0)	69 (23.0)	3.44	1.10
Choice of appointment times	20 (6.7)	19 (6.3)	93 (31.0)	60 (20.0)	108 (36.0)	3.72	1.20
The preparation for your specific test/exam were adequately explained	12 (4.0)	15 (5.0)	102 (34.0)	61 (20.3)	110 (36.7)	3.81	1.11
Registration process at the front desk/courtesy of staff	14 (4.7)	22 (7.3)	56 (18.7)	82 (27.3)	126 (42.0)	3.95	1.15
Explanation of the billing process and procedure	15 (5.0)	27 (9.0)	69 (23.0)	69 (23.0)	120 (40.0)	3.84	1.19
Waiting time before procedure	20 (6.7)	81 (27.0)	83 (27.7)	74 (24.7)	42 (14.0)	3.12	1.15
Courtesy of the nurse/radiographer	8 (2.7)	16 (5.3)	65 (21.7)	62 (20.7)	149 (49.7)	4.09	1.08
Explanation of what to expect during the exam	12 (4.0)	25 (8.3)	159 (53.0)	69 (23.0)	35 (11.7)	3.30	0.92
How questions were answered by the staff	8 (2.7)	9 (3.0)	127 (42.3)	78 (26.0)	78 (26.0)	3.70	0.98
Explanation of what to expect after the exam	18 (6.0)	23 (7.7)	187 (62.3)	46 (15.3)	26 (8.7)	3.13	0.90
The level of attention provided by the nurse/radiographer	8 (2.7)	11 (3.7)	63 (21.0)	85 (28.3)	133 (44.3)	4.08	1.02
The physical appearance of the facilities and the quality of the equipments	8 (2.7)	18 (6.0)	98 (32.7)	95 (31.7)	81 (27.0)	3.74	1.01
What is your overall satisfaction of care received	9 (3.0)	13 (4.3)	51 (17.0)	101 (33.7)	126 (42.0)	4.07	1.02
Overall Mean Score						3.69	
Z-Value						1.983	
*p*-value						0.001	

The overall mean score of the respondents is 3.69. This indicates that on a general note, the respondents were satisfied with the radiological services they received. This result is significant as the Z-value of 1.983 is greater than the critical Z-value of 1.68 (two-tailed test) and p < 0.05. Therefore, the conduct of staff in the department affected the level of patients’ satisfaction.

The activities of radiology nurses which contribute to the overall patient satisfaction in the department are supervised by the radiographer whom the nurses are accountable to.

The conduct of staff that would be more predictive of patient satisfaction would be the ones with the highest mean scores. The conduct of staff that have the most influence on patient satisfaction are courtesy of staff (mean score = 4.09), level of attention provided by the staff (mean score = 4.08) and the patients’ overall satisfaction level stood at mean score of 4.07.

### Assessing for differences and similarities in patients’ satisfaction with ethical conduct of radiographers, their professionalism and skill at both radiology centres

Table 5 shows that the overall patients’ satisfaction with the public radiology centre was 44.31 ± 10.51 while satisfaction with the private radiology centre was 51.45 ± 6.72. A significant difference exists as shown in Table 6 between the satisfaction level patients experienced at these two centres, t (242.13) = −6.960, p < .001, two tailed; with satisfaction at the private radiology centre being higher.

**Table 5:** Descriptive statistics of patients satisfaction level at the two radiology centres

	Radiology centre	N	Mean	Std. deviation	Std. error mean
Satisfaction	public	145	44.3103	10.50761	.87261
	private	155	51.4516	6.72000	.53976

**Table 6:** Comparison of patients satisfaction level at the two radiology centres

		Levene's test for equality of variances		t-test for equality of means						
		F	Sig.	t	df	Sig. (2-tailed)	Mean difference	Std. error difference	95 % C. I of the difference	
									Lower	Upper
Satisfaction	Equal variances assumed	30.153	.000	−7.058	298	.000	−7.14127	1.01176	−9.13237	−5.15016
	Equal variances not assumed			−6.960	242.130	.000	−7.14127	1.02606	−9.16240	−5.12013

According to Table 7, the patients’ assessment of radiographer’s professionalism at the public radiology centre was 34.79 ± 10.51 while at the private radiology centre, their assessment was 36.74 ± 13.20. No significant difference according to Table 8 exists between the professionalism assessment made about radiographers at both centres, t (298) = −1.084, p = .279, two tailed.

**Table 7:** Descriptive statistics of professionalism assessment score of radiographers at the two centres

	Radiology service center	N	Mean	Std. deviation	Std. error mean
Professionalism	public	145	34.7862	17.74792	1.47388
	private	155	36.7355	13.19931	1.06019

**Table 8:** Comparison of professionalism assessment score of radiographers at the two centres

		Levene's test for equality of variances		t-test for equality of means						
		F	Sig.	t	df	Sig. (2-tailed)	Mean difference	Std. Error difference	95 % C. I of the difference	
									Lower	Upper
Professionalism	Equal variances assumed	3.789	.053	−1.084	298	.279	−1.94928	1.79819	−5.48805	1.58949
	Equal variances not assumed			−1.074	265.184	.284	−1.94928	1.81558	−5.52407	1.62552

At the public radiology centre (Table 9), the patients’ assessment of radiographers’ skill was 12.88 ± 7.98 while at the private radiology centre, their assessment was 13.97 ± 7.08. No significant difference (Table 10) exists between the skill assessment made about radiographers at both centres, t (298) = −1.263, p = .208, two tailed.

**Table 9:** Descriptive statistics of skill assessment score of radiographers at the two centres

	Radiological centre	N	Mean	Std. deviation	Std. error mean
Skill	Public	145	12.8759	7.97817	.66255
	Private	155	13.9742	7.07836	.56855

**Table 10:** Comparison of the skill assessment score of radiographers at the two centres

		Levene's test for equality of variances		t-test for equality of means						
		F	Sig.	t	df	Sig. (2-tailed)	Mean difference	Std. error difference	95 % C.I of the difference	
									Lower	Upper
Skill	Equal variances assumed	.123	.726	−1.263	298	.208	−1.09833	.86958	−2.80963	.61297
	Equal variances not assumed			−1.258	288.088	.209	−1.09833	.87305	−2.81670	.62004

## Discussion

Patient satisfaction measure has become an integral part of hospital management strategies and the desired need for this has been driven by the underlying politics of new public management [1]. Radiographers owe patients ethical and professional obligations in their daily conduct in the hospital settings [9, 10]. They also owe patients respect and for them (patients) to be treated with dignity, privacy and confidentiality [9]. Radiographers are also obligated to ensure a safe working environment and are legally accountable for their professional negligence in practice. Many studies [15–18] in Nigeria on patient satisfaction which we reviewed examined overall patient satisfaction with no particular reference to ethical and professional conduct of radiographers and how that could affect patient satisfaction with radiological examination.

In this study, even though radiographers exhibited an overall excellent ethical conduct and professionalism in service delivery, they were found wanting and deficient in many areas: informed consent not being observed, radiographers’ not explaining his/her experience, expectation, knowledge and equipment procedure to patients, radiographers’ inability to disclose his/her competence and limitations where appropriate, radiographers’ inability to understand the need to build and sustain professional relationships with patients and the radiographers’ inability to exercise the need to engage patients in planning and evaluation of diagnostics in service delivery. These are tangible ethical procedures radiographers must not ignore in service delivery because they are directly linked to improved patient satisfaction [9, 10]. It was observed that significant difference exists between the satisfaction level patients experienced at these two centres, with satisfaction at the private radiology centre being higher. The study recommends adequate training in ethical conduct and professionalism for radiographers in the Nigerian university system. Patient’s consent to treatment is a fundamental patient right [9, 11]. This study recommends that it is incumbent on radiographers to relax and assure their patients by sharing their professional experiences and carrying patients along during examination by encouraging their involvement in the procedure. More so regular in-service training and seminars are needed within the hospitals in these areas to prepare radiographers on the task of improving patient satisfaction with radiological service within the department.

On staff conduct concerning service delivery within the radiology department, the overall satisfaction of patients was excellent in this study except for the waiting time before procedure, explanation of what to expect during the examination and explanation of what to expect after the examination which are all sources of dissatisfaction. This may suggest a scheduling problem where patients simply present for radiological examination without being scheduled or even when they are scheduled, there could have been a complete disregard to order as patients may force their way sometimes to see providers before their turn. Yet there could have been insufficient number of radiographers which may prolong patient waiting time unnecessarily. Time management and proper scheduling procedure is recommended. More so, some patients may be confused with the huge machines and procedure of radiology and need to be carried along on what to expect before and after the examination. It is incumbent on the staff to prepare patients for examination [13]. Improving patient satisfaction with radiology will require that staff be trained in customer relations and care which is a recommendation of this study. A formal programme and a body to implement patient satisfaction are recommended for improving patient satisfaction in the radiology departments of hospitals in the locality. More so, it is also recommended that the public health facility in this study learn from the private hospital on what it has done well regarding improved overall patient satisfaction with radiology service.

### Strength and weaknesses of this study

Our study was strengthened by the fact that we improved the calculated sample size from 111 to 300 increasing on the reliability of the study and more so because most of the respondents 142(47.3 %) (Table 1**)** are 30 years of age and below and have between high school and University education meant they could read and write which improved the reliability of the generated data. At least one third 100 (33.3 %) of the respondents were patients that have had previous radiological investigation in the hospitals surveyed. This increased the chance that they were no new comers to radiological examination and that, improved on the reliability of their assessments.

On the other hand, our study was limited by the fact that only two institutions were studied, thus affecting the reliability of the findings. It would have been more desirable to increase the number of institutions studied. The time frame (March--July) within which the study was done is also a source of concern. It would have been better to extend the time frame to include more subjects which could have improved on the reliability of the study. These factors in effect affected the generalisability of our results and external validity.

## Conclusions

The results of our study have demonstrated that ethical and professional conduct of radiographers do influence patients’ satisfaction and dissatisfaction with radiological examination and so are the services offered in the radiology department by members of staff. This area of health service delivery must be accorded the attention it deserves to continually improve on patient satisfaction.
